# Perceptions of Patients and Nurses about Bedside Nursing Handover: A Qualitative Systematic Review and Meta-Synthesis

**DOI:** 10.1155/2024/3208747

**Published:** 2024-04-29

**Authors:** Huda Anshasi, Zainab Abdullah Almayasi

**Affiliations:** ^1^Department of Nursing, College of Health Sciences, University of Fujairah, Fujairah, UAE; ^2^CRN, Kalba Hospital, Kalba, UAE

## Abstract

**Background:**

Bedside nursing handover is a recognized nursing practice that involves conducting shift change communication at the patient's bedside to enhance communication safety. Understanding the perceptions of both patients and nurses regarding bedside handover is crucial in identifying the key principles for developing and implementing effective bedside handover protocols. However, there is currently a lack of comprehensive evidence that summarizes and evaluates studies focused on qualitative approaches for gaining insights into the perceptions of both nurses and patients.

**Purpose:**

This meta-synthesis review aims to identify, synthesize, and evaluate the quality of primary qualitative studies on the perceptions of patients and nurses about bedside nursing handover.

**Methods:**

A meta-synthesis review was conducted to identify qualitative studies that reported patients and nurses' perceptions about bedside handover using seven electronic databases, including CINAHL, PsycINFO, Embase, Education Database (ProQuest), Web of Science, The Cochrane Library, and PubMed, from January 2013 to November 2023. The authors independently selected reviews, extracted data, and evaluated the quality of included studies using the 10-item JBI Qualitative Assessment and Review Instrument tool.

**Results:**

A total of 871 articles were retrieved, of which 13 met the inclusion and exclusion criteria. These studies identified three main themes: (1) facilitators of bedside nursing handover, (2) barriers to bedside nursing handover, and (3) strategies to maintain confidentiality during bedside handover.

**Conclusion:**

This study systematically reviewed and integrated the perceptions of patients and nurses about bedside handover. Based on nurses' perceptions, the combined findings highlight the facilitators of bedside handover, including developing partnership interaction between nurses and patients, promoting professionalism, and enhancing emotional communication among nurses. From the patients' viewpoint, the synthesized findings emphasize the facilitators of bedside handover, including acknowledging the expertise, professionalism, and humanity of the nursing profession, ensuring a sense of safety, satisfaction, and confidence in the care received, as well as promoting individualized nursing care. In the context of barriers to bedside handover, both nurses and patients perceive breaches of confidentiality and privacy violations as significant barriers. When it comes to maintaining confidentiality during bedside handovers, it is important to consider patients' preferences. Patients often prefer handovers to take place in a private setting. From the nurses' perspective, it is important to inquire with patients about their preference for the presence of caregivers, and to conduct private handovers for sensitive issues away from the bedside. *Relevance to Clinical Practice*. Clinicians should carefully evaluate the barriers and facilitators in this meta-synthesis prior to implementing bedside handover. *Study Registration*. This study is registered in PROSPERO with Protocol registration ID: CRD42024514615.

## 1. Introduction and Background

Effective communication is widely regarded as the cornerstone of healthcare systems, particularly within nursing practice [1], where nurses serve as frontline professionals and key players in this intricate environment. In order to effectively practice and fulfill their ultimate mission of “patient care,” nurses must master communication skills [2].

The transfer of patient care, commonly referred to as handoffs or handovers, is a crucial process in healthcare that heavily depends on efficient communication [3]. During handovers, nurses communicate a wide range of information. This includes exchanging patient history, discussing treatments and procedures that need to be completed, and addressing special considerations such as medication allergies, among other important details. Effectively communicating this valuable information is essential for maintaining quality care, patient safety, and treatment continuity during transitions [4].

Nevertheless, numerous obstacles hinder this process, such as heavy workloads, time limitations, stress, critical situations, frequent disruptions, and inevitable noise in the intricate, multidisciplinary healthcare setting. These unavoidable elements can result in communication breakdowns, ultimately affecting patient well-being. The Joint Commission International (JCI) has stated that most medical errors arise from communication breakdowns during handovers [5]. This failure to communicate can result in serious consequences, including medication errors, misdiagnosis, delayed treatments, unnecessary procedures and tests, prolonged hospital stays, and increased costs [4, 6].

Handovers are typically conducted at nursing stations, which are consistently busy areas with frequent noise, including loud voices, alarms, beepers, and phone rings. As a result, distractions and interruptions can easily occur during the exchange of information. These disruptions significantly impact the handover process and contribute to its inefficiency.

To address this issue, bedside handover was introduced as a method to enhance focus, reduce errors, and promote patient safety and patient-centered care [7–10]. By involving the patient in their treatment plan and allowing them to listen to the exchange of information about their condition, they become more aware of their illness [11]. This involvement promotes their empowerment within interprofessional healthcare teams [12], ultimately leading to improved treatment compliance and prognosis. Furthermore, bedside handover leads to improved patient and family satisfaction, nursing quality, and patient safety compared to the traditional hand-off conducted outside the patient's room [13].

Despite the well-established benefits of bedside handoff, healthcare professionals in numerous hospitals still conduct handoffs in the traditional manner, away from the patient's bed [10]. Similarly, many hospitals worldwide persist in utilizing the traditional method of handoff, which can elevate the risk of preventable errors. It is essential for decision-makers to be informed by the latest evidence when considering the implementation of a new policy in this regard.

Exploring and gaining a better understanding of patients' and nurses' perceptions about bedside handover can help identify the principles that should be considered when designing and implementing bedside handover protocols, with a focus on respectfully considering the preferences of both nurses and patients. Numerous qualitative studies have already explored the perceptions of nurses and patients regarding bedside handover in different clinical settings including acute care, medical-surgical ward, emergency department, maternity, and cardiothoracic surgery [14–19]. Despite numerous studies on this topic, only one meta-synthesis has been conducted to evaluate and summarize the existing evidence, specifically focusing on patients' perceptions [20]. To the best of our knowledge, no meta-synthesis has been conducted specifically focusing on the perceptions of both patients and nurses. Furthermore, some new studies have been conducted since the publication of the aforementioned meta-synthesis [11, 14, 21].

Therefore, this meta-synthesis aimed to identify, synthesize, and evaluate the methodological quality of primary qualitative studies on patients' and nurses' perceptions of bedside nursing handover. This meta-synthesis can be a useful tool to aid clinicians, policymakers, and developers of clinical guidelines in making informed decisions. In addition, it helps identify knowledge gaps in the literature and formulates recommendations to enhance the methodological quality of future research in this area.

## 2. Methods

The meta-synthesis was conducted and reported according to the Enhancing Transparency in Reporting the Synthesis of Qualitative Research (ENTREQ) Statement guidelines and Joanna Briggs Institute's (JBI) method of meta-aggregation [22, 23]. The rationale for using the meta-aggregation approach is its ability to generate generalizable recommendations for policymakers [24].

This meta-synthesis was also conducted following the guidelines outlined by Preferred Reporting Items for Systematic Reviews and Meta-Analyses (PRISMA) [25]. The protocol of this meta-synthesis was registered in PROSPERO (CRD42024514615). No amendments were made to the protocol after its registration. The PRISMA/ENTREQ checklist is exhibited in Supplementary File 1.

### 2.1. Research Question

The research question is formulated using the population, exposure, and outcomes (PEO) criteria [26]: what evidence exists regarding the perceptions of patients and nurses about bedside nursing handover?

### 2.2. Search Strategy

The search strategy to identify relevant studies included key terms of “handover,” “handoff,” “nursing handover,” “nursing handoff,” “patient handoff,” “patient handover,” “patient transfer,” “sign out,” “inter shift,” “shift report,” “change of shift,” “shift change,” “service change,” “transition of care,” “bedside report,” “bedside handover,” “patient round,” “shift-to-shift handover,” and “shift-to-shift handoff.” In addition to other terms relevant to the bedside setting, including “patient's room,” “patient participation,” “patient-centered care,” “nurse-patient relations,” and “bedside.” A comprehensive search was conducted to find primary qualitative articles on patients' and nurses' perceptions of bedside nursing handover, utilizing a three-step search strategy. First, a preliminary search was conducted using multiple electronic databases including CINAHL, PsycINFO, Embase, Education Database (ProQuest), Web of Science, The Cochrane Library, and PubMed. After that, an analysis of the text words in the title and abstract, as well as the index terms used to describe the article was conducted. Then, another search was conducted utilizing the previously identified keywords and index terms across all databases included in the study. Then, the relevant articles were sought by examining the reference lists of the included studies. In addition, the Boolean operators (AND/OR) were applied to join the search terms in each database as required.

### 2.3. Inclusion Criteria

Inclusion and exclusion criteria were defined before initiating the search for studies. The PEO (population, exposure, and outcomes) framework was used to develop the basis of the literature search strategy [26]. The primary qualitative studies were selected for this meta-synthesis, if they met the following inclusion criteria: (1) in terms of population, studies including adult nurses and/or adult patients aged 18 years or above. All nurses, including registered nurses and enrolled nurses, were considered, regardless of their professional qualifications; (2) in terms of exposure, studies conducted in any hospital settings where patients and nurses have been exposed to bedside nursing handover; (3) in term of outcomes, studies that presented the perceptions of nurses and/or patients regarding bedside nursing handover in a hospital setting; (4) being published as a full-text research in peer-reviewed journals between November 2013 and November 2023; and (5) published in English language. Excluded from consideration were articles that did not specifically address bedside nursing handovers, multidisciplinary handovers, or the discussion of caregivers' perceptions.

### 2.4. Screening and Selection of Included Studies

All duplicates were removed from the references. The authors (AH and AZ) independently screened the identified study abstracts for potential eligibility in this meta-synthesis. Then, the full-text research was retrieved and carefully examined in detail to assess their eligibility and review relevant outcomes. The authors resolved disagreements by discussing the articles in question until reaching a consensus.

### 2.5. Quality Assessment of the Included Studies

The 10-item JBI Qualitative Assessment and Review Instrument (QARI) [27] software was used to evaluate the methodology quality of eligible qualitative studies. Each item in this tool is categorized into a standardized set of four possible responses: yes, no, unclear, or not applicable. Two authors independently appraised the quality of the studies, and any disagreements were resolved through consensus.

### 2.6. Data Extraction

The data were independently extracted by the two authors, focusing on the authors' names, publication years, study countries, phenomena of interest, participant characteristics, data collection and analysis methods, and the main results. The authors' disagreements were resolved through discussion. No data were requested from the included study's authors.

### 2.7. Data Synthesis

The JBI Qualitative Assessment and Review Instrument (QARI) software was utilized to analyze and categorize findings as either unequivocal (supported by irrefutable data), credible (plausible within the context of the data), or not supported [28].

The findings, which were both “unequivocal” and “credible,” were combined and organized into categories that shared similar meanings. In line with the JBI approach, a category was formed based on a minimum of 2 findings ((JBI), 2020). Inductive thematic content analysis was then performed to analyze the narratives. Two authors (AH and AZ) independently evaluated the categories created, and any disagreements were resolved through consensus.

In order to validate the credibility of the findings in this meta-synthesis, the synthesized results underwent a confidence assessment using the ConQual approach [29]. Created to aid clinical decision-making, this assessment approach categorizes the confidence level of qualitative evidence as high, moderate, low, and very low. All studies commence with a “high” ranking on a scale of high, moderate, low, to very low. Subsequently, they are downgraded depending on their dependability and credibility.

## 3. Results

### 3.1. Study Search and Screening Results

**Figure 1:**
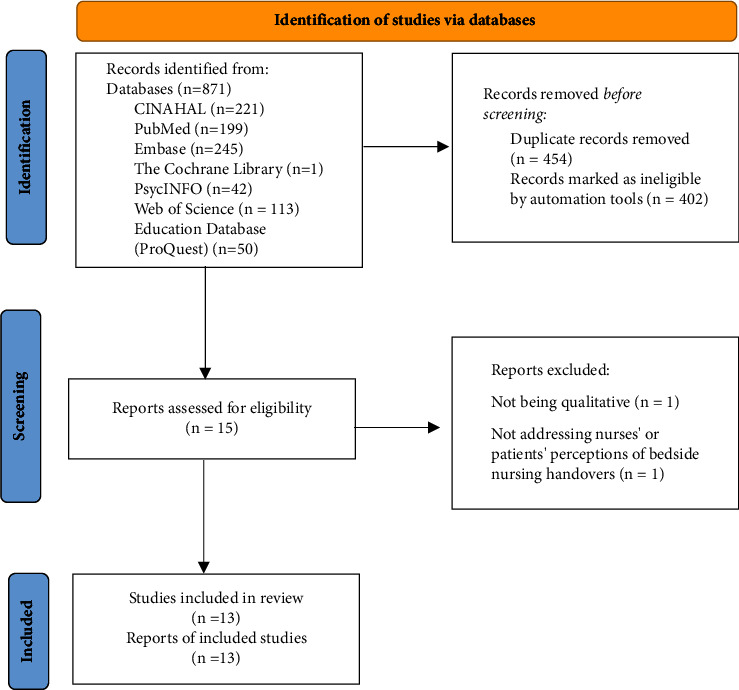
Flowchart of the searching and selecting process.

The search strategies produced 871 records, with 454 duplicates being identified and removed. After reviewing the titles and abstracts, the authors excluded 402 studies and proceeded to assess the full texts of the remaining fifteen studies. Two studies were not included due to one not being qualitative [30] and the other not addressing nurses' or patients' perceptions of bedside nursing handovers [31]. Finally, a total of thirteen studies met the inclusion criterion [11, 13–19, 21, 32–35].  Figure 1 illustrates the flowchart of the searching and selecting process.

### 3.2. Characteristics of Included Studies

The thirteen included studies were published between 2013 and 2021, and 7 (77.8%) of them were published after 2016. Six of these studies were conducted in Australia, three in Italy, two in the US, and the remaining four in Canada, Malaysia, Sweden, and Singapore. Ten of the studies included in this meta-synthesis examined nurses' perceptions [11, 13–16, 18, 19, 21, 33, 35], while three specifically investigated the perceptions of patients [17, 32, 34]. The participants, consisting of 383 nurses and 89 patients, were from various clinical settings including acute care, medical-surgical ward, emergency department, maternity, and cardiothoracic surgery. The characteristics of these twelve included studies are presented in Table 1

### 3.3. Methodological Quality of the Included Studies

All the included studies demonstrated congruity between the research methodology and the research questions, methods used to collect data, analysis, representation, and the conclusions from the data. However, all the included studies failed to clearly demonstrate alignment between the stated philosophical perspective and the research methodology. Furthermore, there is a lack of clear investigation into the influence of the researcher on the research, and vice versa. The authors' agreement on the study qualification ranged from 93% to 100% for each item, resulting in a high overall agreement of 95%. The study's quality details are outlined in Table 2.

The three themes received a moderate evidence quality score because the credibility was downgraded by one level. The details of the level of confidence of the included qualitative studies are presented in Table 3.

### 3.4. Results of the Included Studies

Thirteen studies were conducted to investigate the perspectives of patients or nurses on bedside handover [11, 13–19, 21, 32–35]. The meta-synthesis of thirteen studies resulted in the identification of three main themes: facilitators of bedside nursing handover, barriers to bedside nursing handover, and strategies to maintain confidentiality during bedside handover. These themes encompass fourteen subthemes based on the perceptions of both patients and nurses. The themes and subthemes are shown in Table 4.

#### 3.4.1. Theme 1: Facilitators of Bedside Nursing Handover

*(1) Patients' Perceptions*. The synthesized findings on the facilitators of bedside handover have three subthemes from patients' perceptions which include the following:

  (i) Acknowledging the expertise, professionalism, and humanity of the nursing profession
  This subtheme highlights the shift in patients' perception of the nursing profession, as they increasingly recognize the knowledge, skill, and professionalism demonstrated by nurses. Patients have expressed feeling empowered by the nurses' in-depth understanding of their status and the care plan discussed during bedside handovers [32].

*“It also makes me more aware of what's going on in my health care. Sometimes they change things or do things throughout the day that maybe I'm not aware of, or maybe I am aware of it but didn't really pay attention when they were doing it. This just kind of bumps me up to speed”* ([32], p. 152).

  Furthermore, the patients express gratitude for the humanity displayed by nurses during the information-sharing process at the bedside, which fosters a deeper connection between the nurses and patients [17].

*“I think that, as usual, human beings make the difference. I mean, for me the most important thing is to rely on a high-levelled staff, both defined by professionalism and humanity”* ([17], p. 32).

  (ii). Promoting individualized nursing care
  Patients perceived that bedside handover improved their individualized nursing care. This was due to the opportunity it provided for patients to clarify and give nurses extra information during the verbal handover exchange. Patients viewed bedside nursing handover as a chance to inquire, contribute to their care, and rectify any information with the incoming and outgoing nurses [34].

*“I think it's good. I think it's needed, because if there is something that's wrong, you can always pipe up (speak out). Just so that they've definitely got an understanding of what has happened to you and make sure that it is correct rather than slightly off-key (incorrect). I think it's good and I prefer it”* ([34], p. 1689).

  (iii) Ensuring a sense of safety, satisfaction, and confidence in the care received
  This subtheme highlights how patients feel less anxious about their care, have a sense of security, and experience greater satisfaction as a result of their participation in the bedside nursing handover. Patients expressed feeling a sense of safety, as they were able to promptly verify information during the bedside handover [17]. In addition, the bedside handover provided reassurance to patients that everything was under control, especially during shift changes: *“I felt protected, safe”* ([17], p. 33).
  Patients emphasized the importance of continuity of care. They preferred knowing that all their relevant information was being communicated to the nurses of the next shift. After participating in bedside handover, they felt assured that the transition of care was effectively managed between shifts.

*“So, it's good for me to hear what they've been doing to me, so the next person knows exactly what's been going on. So, it's a smooth transition so, I wouldn't have to re-explain myself again”* ([34], p. 1689).

*(2) Nurses' Perceptions*. The synthesized findings on the facilitators of bedside handover have two subthemes from nurses' perceptions which include the following:

  (iv) Developing partnership interaction between nurses and patients
  This subtheme highlights a key facilitator that is consistently mentioned in the majority of studies: the development of a partnership interaction between nurses and patients during bedside handover [11, 33, 35]. This partnership ensures a safer care process and enhances confidence in the care provided. Nurses perceived that bedside handover increased patients' awareness of their care and strengthened their connection with the nursing staff, enabling them to actively participate in their own care [11]. Bedside handover also provided an avenue for patients to voice concerns about care and allowed nurses to confirm the completeness of the information exchanged, enabling prompt adjustments when necessary [16, 18, 21].
  (v) Promoting professionalism and emotional communication among nurses
  This subtheme highlights another commonly cited facilitator: the promotion of professionalism and emotional communication among nurses during bedside handovers. Nurses have reported that bedside handover promotes the delivery of thorough explanations of clinical information and enables them to communicate with confidence using appropriate language [17]. In addition, nurses have reported that observing their colleagues during bedside handover has provided them with valuable opportunities for mutual learning and teaching, thus strengthening the relationships among the nurses involved in planning and care [16, 21].

#### 3.4.2. Theme 2: Barriers to Bedside Nursing Handover

*(1) Patients' Perceptions*. The synthesized findings on the barriers of bedside handover have two subthemes from patients' perceptions which include the following:

  i) Breaching confidentiality and violating privacy from patients' perceptions
  From the patients' perspective on the violation of their privacy and confidentiality, there is a paradox concerning confidentiality. While patients view respect for privacy as fundamental, most do not express concern during bedside handovers [17]. However, some patients consider their medical information sensitive, especially details about sexually transmitted diseases or addiction, which they perceive as discriminatory. As a result, they prefer that these issues not be discussed during bedside handover [17, 34].

*“Nurses should be sensitive when discussing gynecology issues that should be a bit more private”* ([34], p. 1690).

  Some patients have raised concerns with the volume of nurses' communication during the bedside nursing handover, the disclosure of their full name, and the sharing of information about their condition [32].

*“To be honest, I think something like that (bedside nurse handover) would probably phase out or disappear over a course of 2 days, because after a day or 2 you've heard their bed report, they have heard your bed report”* ([32], p. 152).

  (ii) Neglecting and excluding the patient during bedside handover
  This subtheme highlights the patients' feelings of being excluded or neglected by nurses during the bedside handover process. Patients have described feeling left out due to the use of medical jargon, lack of involvement of the patient during handover, and insufficient attention paid to the fact that the patient is listening [17, 34].
  The use of medical jargon or complex medical language often left patients feeling excluded from important conversations, even when the discussions directly related to their well-being [17]. It appeared that patients were frequently uninformed about their own medical circumstances, despite their belief in their right to understand their diagnosis and treatment plan [17].
  Patients prefer to be more involved during the process, as nurses often talked to each other neglecting that the patient was listening to them: *“they don't tell you anything; I get that they have to talk to one another, but they should involve the patient if there's something concerning him”* ([17], p. 32).

*“They should have answered my questions well, they answered actually but they generally don't pay too much attention to the fact that the patient is listening and would like to know something while they report”* ([17], p. 32).

  Furthermore, patients have occasionally expressed concerns about their understanding during handover, which can lead to anxiety. This problem may be particularly significant for patients whose first language is not English.

*“Well, I understand some things-just simple things. But in very few words-if [nurses] talk too many words, I don't know what (they are) talking about”* ([34], p. 1690).

*(2) Nurses' Perceptions*. The synthesized findings on the barriers of bedside handover have three subthemes from nurses' perceptions which include the following:

  (iii) Breaching confidentiality and violating privacy from nurses' perceptions
  This subtheme reflects the nurses' perspective on the violation of patients' privacy and confidentiality. This is considered a significant obstacle to bedside handover, as identified in the majority of included studies [13, 16, 18, 21].
  Nurses have identified the handling of confidential information as an added layer of complexity during bedside handover [16, 21, 33]. They expressed worries about keeping patient information confidential during bedside handover, particularly when it relates to private (e.g., cancer and palliative care) or sensitive (e.g., blood-borne virus infection and drug and alcohol history) matters [33].
  They also expressed significant concern about the potential privacy violations caused by the presence of other patients or caregivers [13, 16, 18, 21].

*“I feel there's a privacy issue, saying everything out loud. I try to speak quietly but curtains aren't soundproof. I don't like that”* ([33], p. 254).

  (iv) Rising risk of overtime and unnecessary transmission of redundant information
  This subtheme brings attention to the concerns raised by nurses in many included studies regarding the potential for increased overtime and the transmission of redundant information during bedside handover [13, 16, 18, 35]. Nurses who are required to work extra hours may feel pressured to hurry to finish all their duties, including bedside handover [13, 16, 18]. Furthermore, patients involved in bedside handover with nurses working overtime may feel frustrated, perceiving the nurses as absent, superficial, and lacking full involvement in the therapeutic relationship [35].
  Nurses have reported that distractions, noises, and interruptions are unavoidable during bedside handover, and that these factors may potentially result in omissions [19, 21]. They believe that the traditional handover method, where nurses gathered around a table, posed less risk of information being overlooked [18, 21].The necessity to repeatedly convey the same information at every shift can be overwhelming for both nurses and patients.
  Nurses held varying opinions about the importance of patient involvement in bedside handover [19]. Some argued that patients should be excluded from this process because they lack expertise, and nurses often experience fatigue when trying to understand patients' needs, which can be time-consuming [35]. The occurrence of such failures has caused nurses to conduct superficial and abbreviated bedside handovers [35], resulting in patients feeling left out and creating negative views of the process [17].
  (v) Contribute to decrease in the sense of collegiality and security
  This subtheme highlights a common barrier identified in many studies, where nurses perceive that bedside handover may lead to frustrations among colleagues, as well as feelings of uncertainty and discomfort [13, 16, 19]. Nurses explicitly referred to the freedom of expression provided by nonbedside handover methods, which were dedicated to sharing concerns and alleviating frustrations among colleagues [19]. In situations where nurses with lower skill levels and fewer patients to care for, implementing bedside handover often resulted in demands to revert to previous handover methods [13]. Bedside handovers occasionally caused nurses to feel uncertain, particularly if they sensed judgment from patients or coworkers [16]. Nurses have expressed that being interrogated in front of the patient without adequate information is a source of anxiety and can lead to embarrassing situations [16, 18]. At times, nurses have expressed that engaging in an intellectual activity such as bedside handover, which lacks a technical component, has led to feelings of embarrassment. This is due to patients not perceiving bedside handover as an essential procedure, but rather as a casual conversation among staff that does not align with the standard of care [18].
  Moreover, nurses have described that bedside handover may lead to feelings of uncertainty and discomfort. Nurses have conveyed a feeling of uncertainty regarding the appropriate level of detail to be utilized during bedside handover [11]. They specifically experienced difficulty in responding to patients' inquiries during bedside handover and expressed frustration regarding the potential confusion it could cause [16]. Communication issues were particularly prevalent among novice nurses [21]. Nurses perceive bedside handover as a means for the staff to exert control and assert authority over them [17].

#### 3.4.3. Theme 3: Strategies to Maintain Confidentiality during Bedside Handover

*(1) Patients' Perceptions*. The synthesized findings on the strategies to maintain confidentiality during bedside handover have two subthemes from patients' perceptions which include the following:

  (i) Nurses should use discretion when handling sensitive issues
  According to patients' perceptions, nurses are expected to exercise discretion when handling sensitive information during bedside handovers, including topics such as sexual health and alcohol issues, in order to maintain confidentiality. The discussion included various methods for nurses to communicate this information during handover, such as speaking softly or relocating away from the patient's bedside [34].

*“I think nurses already act discreet(ly) with sensitive issues and don't vocalize it for everyone to hear”* ([34], p. 1690).

  Patients anticipate that nurses will handle new or distressing information professionally. They believe that sensitivity is necessary when patients are unaware of the information about their condition, indicating that such news should not be conveyed during the nurses' handover conversation [34].
  Patients typically found satisfaction in acquiring information, while nurses were required to convey it with professionalism, empathy, and compassion, particularly when delivering unfavorable news [17].

*“The positive is always positive, while the negative gets you down; you perceive there's something wrong”* ([17], p. 33).

  (ii) Preference for handover to occur in the cubicle
  This subtheme highlights another strategy to maintain confidentiality during handover. Many patients reported expressed worry about nurses discussing their condition in areas where others might overhear, possibly violating their confidentiality [34].

*“The only bad thing is that other patients can hear when they do it outside the cubicle”* ([34], p. 1690).

*“I prefer nurses to talk about personal information here in the cubicle not outside where other patients can hear”* ([34], p. 1690).

*(2) Nurses' Perceptions*. The synthesized findings on the strategies to maintain confidentiality during bedside handover have two subthemes from nurses' perceptions which include the following:

  (iii) Inquiring with patients about their preference for the presence of caregivers
  Nurses in the included studies have discussed various strategies for upholding confidentiality during bedside handover. These strategies encompass asking patients about their preference for the presence of caregivers [15, 21], indicating information in the patient's chart, and requesting visitors to leave the room before discussing sensitive details [33].
  In addition, ensuring a private setting enhanced confidentiality during bedside handover [35], as did the pacts between nurses and patients concerning the information shareable in the company of others [18, 34].

*“We ask the patient if they are alright if we give a handover while they (visitors, family) are there and if they are not comfortable then we can always say to them” “if you can step out of the room (referring to visitors and family) so I can give handover”* ([15], p. 124).

  (iv) Nurses should conduct private handovers for sensitive issues away from the bedside
  The nurses in the studies included have emphasized the importance of discussing sensitive issues away from the bedside and conducting private handovers. Nurses seek flexibility in managing confidential health information, as indicated by the following comments:

*“Small things will be (discussed) out in the station; all the confidentiality issues are all discussed in front of our big board. If we've got to say something like “these patients got HIV,” “they've got cancer;” we can do that discretely in another forum”* ([15], p. 124).

## 4. Discussion

This meta-synthesis examined the findings of qualitative studies exploring patients' and nurses' perspectives of bedside handover in a hospital setting. In order to ensure that this meta-synthesis is informative for clinicians and researchers, the authors established inclusive criteria to encompass all qualitative evidence regarding the perceptions of bedside handover from the perspectives of both nurses and patients. They then categorized the data based on the participants.

### 4.1. Discussion of the Themes Emerged

The meta-synthesis identified three key themes: facilitators of bedside nursing handover, barriers to bedside nursing handover, and strategies to maintain confidentiality during bedside handover. These findings have the potential to enhance our understanding of nurses' and patients' perceptions regarding bedside handover. The synthesized findings exhibit a moderate level of confidence, indicating their suitability for incorporation into clinical practice.

In terms of methodological quality, improvements should be made in the following respects: (1) ensuring alignment between the stated philosophical perspective and the study methodology, (2) clearly stating the cultural or theoretical background of the researcher, and (3) addressing the influence of the researcher on the research, and conversely.

#### 4.1.1. Theme 1: Facilitators of Bedside Nursing Handover

This meta-synthesis highlights the facilitators of bedside handover from the perspective of nurses. These include the development of a partnership interaction between nurses and patients, as well as the promotion of professionalism and emotional communication among nurses. These findings align with other studies that have shown a positive correlation between bedside handovers and increased staff interaction, as well as improved patient communication [36–38]. The findings indicate the importance of implementing bedside handover to improve patient communication, thereby ensuring a safer care process.

From patients' perceptions, the synthesized findings on the facilitators of bedside handover include ensuring a sense of safety, satisfaction, and confidence in the care received. The findings align with previous studies demonstrating that bedside handover improves the nursing handover process, elevates patient satisfaction, and enhances patient safety [39, 40].

The promotion of individualized nursing care is another key facilitator of bedside handover, as perceived by patients. These findings are consistent with previous studies that have highlighted the enhancement of patient-centered care through bedside handover [7]. The findings of this meta-synthesis suggest that clinicians should consider implementing bedside handover, taking into account the facilitators identified from the perspectives of both nurses and patients.

#### 4.1.2. Theme 2: Barriers to Bedside Nursing Handover

Based on the findings of this meta-synthesis, both nurses and patients perceive breaching confidentiality and violating privacy as significant barriers to bedside handover. Both nurses and patients express varying levels of concern about these issues, with nurses showing a greater concern for confidentiality compared to patients. Nurses consider threats to patients' confidentiality as the primary obstacle to implementing bedside handover [13, 16, 18, 21]. They expressed worries about keeping patient information confidential during bedside handover, particularly when it relates to sensitive (e.g., alcohol history) matters [33]. This aligns with findings from some studies included in this meta-analysis, which have highlighted that some patients view their medical information, particularly details about sexually transmitted diseases or addiction, as sensitive and potentially discriminatory [34, 41]. Consequently, they prefer that these issues not be addressed during bedside handovers. These findings suggest that sensitive information such as sexually transmitted diseases or addiction should not be discussed during bedside handover. Clinicians should consider this barrier prior to implementing bedside handover.

Based on patients' perceptions, another barrier to bedside handover, as indicated by the findings of this meta-synthesis, is the neglect and exclusion of the patient during bedside handover. The use of medical jargon or complex medical language often left patients feeling excluded from important conversations, even when the discussions directly related to their well-being [17, 34]. The findings align with a meta-synthesis that confirmed how unfamiliar language can restrict patients' comprehension of information exchanged during handover, consequently impacting their active participation and perceptions [20]. Therefore, the findings of this meta-synthesis highlight the significance of nurses using simple and comprehensible language when communicating with patients during handovers. It is important for nurses to avoid using medical jargon and to explain terms in a manner that patients can easily grasp.

#### 4.1.3. Theme 3: Strategies to Maintain Confidentiality during Bedside Handover

The theme explored nurses' and patients' strategies for maintaining confidentiality during bedside handover. According to patients' perceptions, nurses are expected to exercise discretion when handling sensitive information during bedside handovers, including topics such as sexual health, in order to maintain confidentiality. The discussion included various methods for nurses to convey this information during handover, such as speaking softly or relocating away from the patient's bedside [34]. These findings are consistent with nurses' perceptions, and they have emphasized the importance of discussing sensitive issues away from the bedside and conducting private handovers. Clinicians should consider this strategy prior to implementing bedside handover.

Other strategies for maintaining confidentiality during bedside handover, from the perspective of nurses, include inquiring about patient preferences regarding caregiver presence [15, 21], recording information in the patient's chart, and asking visitors to leave before discussing sensitive details [33]. Nurses need to be ready to be flexible in how they conduct the bedside handover. Understanding the importance of being flexible during bedside handovers is crucial for their successful implementation.

Therefore, the findings of this meta-synthesis indicate the importance of implementing an educational program for patients and nurses regarding the process of conducting and participating in bedside handover. The educational program for patients aims to deliver clear instructions to them regarding their role and the purpose of bedside handover. This involves notifying them about the handover timing, the participants, the discussion topics, and how they can actively engage in the process [35]. The educational program for nurses aims to educate them about the importance and benefits of involving patients in handover procedures before conducting bedside handovers. Comprehending the significance of bedside handover can inspire nurses to actively engage patients during handovers. One of the included studies recommends integrating active educational activities for nurses, such as simulations that simulate real-life scenarios. These simulations offer nurses the opportunity to reflect on and cultivate suitable, patient-centered responses [19]. Future research should further evaluate these by integrating active educational activities and other implementation strategies in enhancing bedside handover.

### 4.2. Limitations of the Study Findings

While this paper was conducted according to the JBI meta-aggregative approach, which has a strong feature of enabling generalizable recommendations for policymakers, it is noteworthy that the majority of included studies were conducted in Western countries. This may limit the generalizability of the study's findings to other regions, such as Arab countries. Also, the level of care in all included studies was quite heterogeneous.

## 5. Conclusion

This study systematically reviewed and integrated the perceptions of patients and nurses about bedside handover and identified three main themes: facilitators of bedside nursing handover, barriers to bedside nursing handover, and strategies to maintain confidentiality during bedside handover. Based on nurses' perceptions, the combined findings highlight the facilitators of bedside handover, including developing partnership interaction between nurses and patients, promoting professionalism, and enhancing emotional communication among nurses. From the patients' viewpoint, the synthesized findings emphasize the facilitators of bedside handover, including acknowledging the expertise, professionalism, and humanity of the nursing profession, ensuring a sense of safety, satisfaction, and confidence in the care received, as well as promoting individualized nursing care. These findings underscore the importance for clinicians to incorporate these facilitators when implementing bedside handover.

In the context of bedside handover, both nurses and patients perceive breaches of confidentiality and privacy violations as significant barriers. According to nurses, other barriers include the risk of overtime, unnecessary transmission of redundant information, and potential contribution to a decreased sense of collegiality and security. Patients also perceive neglect and exclusion during bedside handover as additional barriers. Clinicians should carefully evaluate these barriers prior to implementing bedside handover.

When it comes to maintaining confidentiality during bedside handovers, it is important to consider patients' preferences. Patients often prefer handovers to take place in a private setting, such as a cubicle. In addition, nurses should use discretion when managing sensitive issues. From the nurses' perspective, it is important to inquire with patients about their preference for the presence of caregivers, and to conduct private handovers for sensitive issues away from the bedside.

## 6. Relevance to Clinical Practice

The findings of this meta-synthesis are highly valuable for clinicians who are looking to implement bedside handover as a way to improve the quality of healthcare. They should carefully evaluate the barriers and facilitators in this meta-synthesis prior to implementing bedside handover. The findings of this meta-synthesis also indicate the importance of implementing an educational program for patients and nurses regarding the process of conducting and participating in bedside handover.

## 7. Implications for Future Research

Further research is required to explore the perceptions of patients and nurses regarding bedside handover, particularly in Arab countries. It is essential to conduct further high-quality methodological research with a specific emphasis on implementation strategies for bedside handover. In terms of methodological quality, improvements should be made in the following respects: (1) ensuring alignment between the stated philosophical perspective and the research methodology, (2) clearly stating the cultural or theoretical background of the researcher, and (3) addressing the influence of the researcher on the research, and vice versa.

## Figures and Tables

**Table 1:** Characteristics of the studies included in the meta-synthesis.

Author (year)	Country	Phenomena of interest	Participants characteristics (*n*)	Data collection/data analysis	Main findings/themes
Johnson and Cowin (2013) [15]	Australia (Sydney)	To explore how nurses experienced the introduction of bedside handover and the use of written handover sheets	(i) Registered nurses and enrolled nurses (similar to licensed practical nurses)	Focus groups/thematic analysis	“(1) Bedside handover strengths and weaknesses (most participants believed that confidentiality issues arising from handovers were minimal and easily managed within their current handover practice although permission is sometimes sought from patients and significant others); (2) patient involvement in handover; (3) good communication is about good communicators; and (4) three sources of patient information (handover, handover sheets, and nursing notes” [15]
			(ii) Sample from medical and surgical wards at three major metropolitan hospitals		
			(iii) Sample size: *n* = 30		
			(iv) Age: 21 yrs—50 yrs		
			(v) Gender: male (5) and female (25)		

Kerr et al. (2013) [34]	Australia	To explore the perspectives of patients about bedside handover by nurses in the emergency department	(i) Patients from the emergency department (ED)	Semistructured interviews/thematic content analysis	“(1) Patients perceive that participating in bedside handover enhances individual care and (2) maintaining privacy and confidentiality during bedside handover is important for patients (preference was expressed for handover to be conducted in the ED cubicle area to protect the privacy of patient information)” [34]
			(ii) Sample size: *n* = 30		
			(iii) Age: ≥18 yrs		
			(iv) Gender: male (12) and female (18)		

Kerr et al. (2014) [33]	Australia	To explore the perspectives of nurses and midwives towards the introduction of bedside handover	(i) Nurses (registered nurse and enrolled nurse) and midwives	Semistructured interviews/content analysis	“(1) Enhanced individual patient care and documentation, along with improved patient-clinician partnerships and (2) protection of confidentiality and privacy (private and/or sensitive information can be communicated by whispering, pointing at the information in the patient's chart, or after asking visitors to leave the room)” [33]
			(ii) Nurses sample from medical and surgical wards		
			(iii) Sample size: registered nurse *n* = 17, enrolled nurse *n* = 3, and midwives *N* = 10		
			(iv) Gender: male (5) and female (25)		

Jeffs et al. (2014) [32]	Canada (Toronto)	To explore patients' experiences and perceptions associated with the implementation of bedside nursing handover	(i) Patients from one acute care hospital	Semistructured interviews/a directed content analysis	“(1) Creating a space for personal connection (patients described bedside nursing handover as an engaging, personal, and informative approach to shift handover); (2) bumping up to speed (patients described feeling less anxious about their care, having a sense of security, and experiencing greater satisfaction as a result of their participation in the bedside nursing handover); and (3) varying preferences (patients emphasized the importance of considering their clinical status as well as the preferences of each individual patient before performing bedside nursing handover)” [32]
			(ii) Sample size: *n* = 45		
			(iii) Age: ≥18 yrs		
			(iv) Gender: male (15) and female (30)		

Grimshaw et al. (2016) [13]	USA	To identify factors and acute care nurses' perceptions influencing the frequency of change-of-shift reports at the bedside	(i) Nurses from medical unit, surgical unit, and intensive care unit (ICU)	Semistructured interviews/thematic analysis	“Five themes were identified from the collected data, which included the time factor, continuity of care, visualization, and challenges in the communication of discreet information” [13]
			(ii) Sample size: *n* = 7		
			(iii) Age: 19 yrs–59 yrs		
			(iv) Gender: male (1) and female (6)		

Lupieri et al. (2016) [17]	Italy	To explore the postoperative cardiothoracic surgical patient experience of nurses' bedside handovers	(i) Patients from a cardiothoracic ICU	Semistructured interviews/Content analysis	“(1) Discovering a new nursing identity (patients getting to know the competence involved in the nursing profession leading to improved patients' satisfaction); (2) being apparently engaged in a bedside handover (limited patients' participation, patients prefer to be more involved); (3) experiencing the paradox of confidentiality (lack of privacy is problem but did not represent a concern for patients and patients do not care if another patient know about their medical problems); and (4) having the situation under control (nurses were able to verify the information immediately and feeling sense of safety)” [17]
			(ii) Sample size: *n* = 14		
			(iii) Age: ≥18 yrs		
			(iv) Gender: male (10) and female (4)		

Khuan and Juni (2017) [35]	Malaysia	To explore Malaysian nurses' opinions about patient involvement in relation to patient-centered care during bedside handovers	(i) Registered nurses from medical, surgical, and orthopedic wards	Focus groups/content analysis	“(1) Superficial involvement related to a knowledge deficit, inexperience, and a task-oriented mindset; (2) patient-centered care (PCC) as interactive and respectful of patients' wishes and/or decisions; (3) impracticality of patient involvement in relation to time constraints, length of interaction, and hierarchy of nurse-patient communication; and (4) patient involvement as not representative of PCC due to violations of patient autonomy” [35]
			(ii) Sample size: *n* = 20		
			(iii) Age: 26 yrs–40 yrs		
			(iv) Gender: male (6) and female (14)		

Roslan and Lim (2017) [18]	Singapore	To explore nurses' perceptions of bedside clinical handover in an acute-care inpatient unit in Singapore	(i) Registered nurses, enrolled nurses, and nurse clinicians from an acute-care hospital	Focus groups/Semistructured interviews/thematic analysis	“(1) Bedside clinical handover could compromise patient's confidentiality (potential confidentiality breach, demands for secrecy, and risk of information misinterpretation) and (2) disturbances during the handover (patient and/or their family members and environment are sources of constant interruptions and distractions)” [18]
			(ii) Sample size: *n* = 20		
			(iii) Gender: male (1) and female (19)		

Tobiano et al. (2017) [19]	Australia	To explore and understand barriers nurses perceive in undertaking bedside handover	(i) Registered nurses, enrolled nurses from medical wards	Open-ended question/content analysis	“(1) Censoring the message showed that nurses were concerned about patients and third parties hearing sensitive information; (2) disrupting the communication flow, nurses perceived patients, family members, other nurses, and external sources interrupted the flow of handover and increased its duration; and (3) inhibiting characteristics demonstrated that individual patient and nurse views or capabilities hindered bedside handover” [19]
			(ii) Sample size: *n* = 176		
			(iii) Gender: male (18) and female (158)		

Dellafiore et al. (2019) [11]	Italy	To explore the perceptions of nurses regarding a recent implementation of bedside nursing handover	(i) Nurses from cardiac surgery	Focus groups/thematic analysis	“The main themes that were identified revolved around improving nursing care, greater professionalism, effective relationships, consequences for the patient, and obstacles to change. Moreover, we found that nurses perceive bedside nursing handover to be effective in promoting patient-centered care. The nurses in our study also felt that any difficulties with the implementation of a bedside nursing handover protocol (e.g., confidentiality) should be addressed through continued nursing education” [11]
			(ii) Sample size: *n* = 16		
			(iii) Age: 26 yrs–48 yrs		

Hada et al. (2019) [21]	Australia (Brisbane)	To systematically assess the barriers and facilitators to evidence-based nursing handover in a clinical environment, and to identify potential adopters and attributes of evidence-based nursing handover for translation into practice	(i) Registered nurses, enrolled nurses from medical wards	Focus groups/content analysis	“(1) Content (information transferred); (2) process (steps used to transfer accountability and responsibility for care); and (3) environment (factors impacting safe handover). Participants identified barriers to effective nursing handover including variability of the handover content and process, uncertainty around sharing sensitive information, inconsistency around clarifying gaps through questioning during the handover, superficial patient involvement, time constraints, and environmental challenges. The key facilitators discussed during the focus groups were the use of integrated electronic medical records, support and clear expectations from the nursing leadership, and targeted handover education” [21]
			(ii) Sample size: *n* = 49		
			(iii) Gender: male (8) and female (41)		

Jimmerson et al. (2021) [14]	USA	Experiences and opinions regarding bedside shift report	(i) Clinical nurses and their nursing supervisors from acute adult care inpatient units	An in-depth face-to-face interview	“(1) Time constraints nurse's workflow (more time is spent making the discussion professional because the patient is hearing, patients' interruptions are time-consuming, and can increase the risk of missing important information); (2) modified approach needed (against full bedside handoff, rehearsal outside patient's room can help, and patient presence restricts the type of information shared); (3) individualization of process and content to be meaningful (does not fit all size, problem lists and management should be the focus, and waking patient up for the handover is not good); (4) specific critical content that should be discussed inside the patient's room (plan of care/goals for day and medication infusion); and (5) specific critical content that should be discussed outside the patient's room (history and review of systems)” [14]
			(ii) Sample size: clinical nurses (*n* = 22) and nursing supervisors (*n* = 12)		
			(iii) Age: ≥18 yrs		

**Table 2:** Methodological quality of the qualitative studies included in the meta-synthesis.

Author (year)	Q1	Q2	Q3	Q4	Q5	Q6	Q7	Q8	Q9	Q10	Overall score
Johnson and Cowin (2013) [15]	U	Y	Y	Y	Y	N	U	Y	Y	Y	7
Kerr et al. (2013) [34]	U	Y	Y	Y	Y	N	U	Y	Y	Y	7
Kerr et al. (2014) [33]	U	Y	Y	Y	Y	U	U	Y	Y	Y	7
Jeffs et al. (2014) [32]	U	Y	Y	Y	Y	N	U	Y	Y	Y	7
Grimshaw et al. (2016) [13]	U	Y	Y	Y	Y	U	U	Y	Y	Y	7
Lupieri et al. (2016) [17]	U	Y	Y	Y	Y	U	U	Y	Y	Y	7
Khuan & Juni (2017) [35]	U	Y	Y	Y	Y	U	U	Y	Y	Y	7
Roslan & Lim (2017)[18]	U	Y	Y	Y	Y	U	U	Y	Y	Y	7
Tobiano et al. (2017) [19]	U	Y	Y	Y	Y	U	U	Y	Y	Y	7
Kulberg et al. (2018) [16]	U	Y	Y	Y	Y	U	U	Y	Y	Y	7
Dellafiore et al. (2019) [11]	U	Y	Y	Y	Y	U	U	Y	Y	Y	7
Hada et al. (2019) [21]	U	Y	Y	Y	Y	Y	U	Y	Y	Y	8
Jimmerson et al. (2021) [14]	U	Y	Y	Y	Y	Y	U	Y	Y	Y	8
Number of Y (%)	0 (0)	13 (100)	13 (100)	13 (100)	13 (100)	2 (15.38)	0 (0)	13 (100)	13 (100)	13 (100)	

Y = yes; N = no; U = unclear. JBI-QARI, Joanna Briggs Institute Qualitative Assessment and Review Instrument (JBI-QARI) critical appraisal tool; Q1. Is there a congruity between the stated philosophical perspective and the research methodology? Q2. Is there a congruity between the research methodology and the research question or objectives? Q3. Is there a congruity between the research methodology and the methods used to collect data? Q4. Is there a congruity between the research methodology and the representation and analysis of data? Q5. Is there a congruity between the research methodology and the representation and analysis of data? Q6. Is there a statement locating the researcher culturally or theoretically? Q7. Is the influence of the researcher on the research, and vice-versa, addressed? Q8. Are participants, and their voices, adequately represented? Q9. Is the research ethical according to the current criteria or, for recent studies, and is there evidence of ethical approval by an appropriate body? Q10. Do the conclusions drawn in the research report flow from the analysis, or interpretation, of the data?

**Table 3:** Level of confidence of the qualitative studies included in the meta-synthesis.

Synthesized finding/themes	Type of study	Dependability	Credibility	ConQual score^*∗*^
Facilitators of bedside nursing handover	Qualitative	Unchanged	Downgrade one level	Moderate
Barriers to bedside nursing handover	Qualitative	Unchanged	Downgrade one level	Moderate
Strategies to maintain confidentiality during bedside handover	Qualitative	Unchanged	Downgrade one level	Moderate

^*∗*^All studies commenced with a “high” ranking on a scale of high, moderate, low, to very low, and then downgraded based on their dependability and credibility.

**Table 4:** Synthesized themes according to the patients' and nurses' perceptions about bedside handover.

Themes	Subthemes/categories
Facilitators of bedside nursing handover	Patients' perceptions
	(i) Acknowledging the expertise, professionalism, and humanity of the nursing profession
	(ii) Promoting individualized nursing care
	(iii) Ensuring a sense of safety, satisfaction, and confidence in the care received
	Nurses' perceptions
	(i) Developing partnership interaction between nurses and patients
	(ii) Promoting professionalism and emotional communication among nurses

Barriers to bedside nursing handover	Patients' perceptions
	(i) Breaching confidentiality and violating privacy from patients' perceptions
	(ii) Neglecting and excluding the patient during bedside handover
	Nurses' perceptions
	(i) Breaching confidentiality and violating privacy from nurses' perceptions
	(ii) Rising risk of overtime and unnecessary transmission of redundant information
	(iii) Contribute to decrease in the sense of collegiality and security

Strategies to maintain confidentiality during bedside handover	Patients' perceptions
	(i) Nurses should use discretion when handling sensitive issues
	(ii) Preference for handover to occur in the cubicle
	Nurses' perceptions
	(i) Inquiring with patients about their preference for the presence of caregivers
	(ii) Nurses should conduct private handovers for sensitive issues away from the bedside

## Data Availability

The data used to support the findings of this study are available from the corresponding author upon request.

## Supplementary Materials

Supplementary Materials This section includes the Enhancing Transparency in Reporting the Synthesis of Qualitative Research (ENTREQ) Statement guidelines and Preferred Reporting Items for Systematic Reviews and Meta-Analyses (PRISMA) checklist.
